# Knowledge, Attitudes, and Practices of Hematologists Regarding Fertility Preservation in Turkey

**DOI:** 10.4274/Tjh.2012.0015

**Published:** 2013-09-05

**Authors:** Mert Küçük, İrfan Yavaşoğlu, Ali Zahit Bolaman, Gürhan Kadıköylü

**Affiliations:** 1 Adnan Menderes University, Faculty of Medicine, Department of Obstetrics and Gynecology, Aydın, Turkey; 2 Adnan Menderes University, Faculty of Medicine, Department of Internal Medicine, Division of Hematology, Aydın, Turkey

**Keywords:** Hematologists, Fertility preservation, Attitude

## Abstract

**Objective:** Fertility preservation stands before us as an issue of quality of life for cancer patients and their partners and families. Therefore, the object of the present study was to determine the extent of the knowledge that hematologists have about fertility preservation and to understand their attitudes and practices regarding this matter.

**Materials and Methods:** A total of 25 hematologists participated in a survey. The questionnaire included questions on sociodemographic characteristics and awareness concerning the subject of fertility preservation, as well as questions designed to determine the extent of the knowledge that hematologists had on the subject and to understand their attitudes and practices in this context.

**Results:** Of the participants in the study, all expressed their awareness of the adverse effects that the various treatments they were prescribing could have on fertility; 2 (8%) revealed that they had never heard of the concept of fertility preservation. Of the participants, 19 (76%) indicated that they did not have adequate knowledge about fertility preservation, but 22 (88%) fortunately expressed a need for acquiring more knowledge about the subject. Of the respondents, 23 (92%) said that they did not have any brochures or published resources on this subject and stated their belief that if hematologists did have such documents, they would have more opportunity to discuss the various fertility preservation options with patients. All of the participants in the survey supported the idea of the Turkish Society of Hematology publishing a guidebook on this subject and organizing a session on fertility preservation in their regular congress.

**Conclusion:** Meeting the needs of hematologists for training and knowledge in the subject of fertility preservation and ensuring the development of appropriate attitudes and practices in this area is an important issue. The Turkish Society of Hematology may play a significant key role.

**Conflict of interest:**None declared.

## Abstract (tr)

**Amaç:** Fertility prezervasyonu önemli bir hayat kalitesi konusu olarak kanserli hastalar, partnerleri ve aileleri için karşımıza çıkmaktadır. Bu nedenle bu çalışmada hematologların fertilite prezervasyonu konusunda ne ölçüde bilgi sahibi olduklarını belirlemek, ve hematologların bu konuda tutum ve davranışlarını anlamak amaçlanmıştır.

**Gereç ve Yöntemler:** Ankete toplam 25 hematolog katıldı. Anket katılımcıların sosyodemografik özellikleri, fertilite prezervasyonu konusunda farkındalıkları kadar hematologların bu konuda bilgilerinin seviyesini belirlemek ve bu bağlamda tutum ve davranışlarını anlamak için sorular içerdi.

**Bulgular:** Katılımcıların tamamı reçete ettikleri çeşitli tedavilerin fertilite üzerine olumsuz etkileri olabileceği konusunda farkındalıkları olduğunu belirtti; iki katılımcı (%8) ise fertilite prezervasyonu kavramını hiç duymadığını ifade etti. Katılımcıların 19’u (%76) fertilite prezervasyonu konusunda yeterli bilgiye sahip olmadığını ifade etti ve şanslı olarak katılımcıların 22’si (%88) fertilite prezervasyonu konusunda daha fazla bilgi almak ihtiyacı olduğunu ifade etti. Katılımcıların 23’ü (%92) ellerinde bu konuda broşür veya basılı kaynak olmadığını ifade etti ve hematologların böyle dökümanları bulunması halinde çeşitli fertilite prezervasyon seçeneklerini tartışmak için daha fazla fırsatları olacağını belirttiler. Katılımcıların tamamı Türk Hematoloji Derneğinin bu konuda bir klavuz basması fikrini desteklediklerini ve düzenli kongrelerinde fertilite prezervasyonu konusunda bir oturum olmasını desteklediklerini belirtti.

**Sonuç:** Hematologların fertilite prezervasyonu konusunda eğitim ve bilgi ihtiyaçlarının karşılanması ve bu konuda uygun tutum ve davranışlarının geliştirilmesinin sağlanması önemli bir husustur. Bu konuda Türk Hematoloji Derneği önemli ve kilit bir rol oynayabilir.

## INTRODUCTION

The advances made in providing effective treatment options and the steadily increasing percentage of cured patients or of patients with 5-year life expectancies in hematological malignancies have brought the subject of such patients’ quality of life into the foreground [1]. In this respect, the quality of life of patients of reproductive age demands particular attention. Studies on fertility or fertility preservation have highlighted the importance of these issues in terms of the quality of life of cancer patients [2,3]. Research indicates that loss of fertility or the fear of a loss of fertility among young cancer survivors is a significant trigger of psychological morbidity [4]. 

With the development of combination chemotherapies and steady improvements in more effective treatment modalities, today the 5-year survival rate of patients with Hodgkin’s lymphoma has reached a level of 90% [5]. It is known, however, that many types of treatment used in Hodgkin’s lymphoma or other hematological malignancies have a gonadotoxic effect [3]. Fertility problems thus appear before us as a major quality of life issue in the case of young patients with this disease [5]. 

Today, the subject of fertility preservation is steadily gaining more and more attention. An increasing number of clinicians are reporting that patients and their families and spouses are fearful about loss of fertility and reporting their will and desire to discuss fertility preservation options. Major associations in the United States have published recommendations on this subject [6,7]. 

Today, sperm and embryo cryopreservation are the first options that are offered in fertility preservation [6,7,8]. Live pregnancies attained with oocyte and ovarian tissue cryopreservation represent the advanced fertility preservation options that are available today. There are many more options that are still in their experimental stages at the moment. 

There are only a limited number of studies in the literature today that have explored the matter of fertility preservation from the standpoint of hematologists. To the best of our knowledge, there has been no study conducted in Turkey on the knowledge, attitudes, and practices of hematologists regarding fertility preservation. This study therefore was carried out to understand how aware hematologists are about the subject of fertility preservation, the extent of their knowledge of this topic, their attitudes toward fertility preservation, and what their practices are in this area as a part of their daily work. 

## MATERIALS AND METHODS

The study was conducted as descriptive and cross-sectional research. An attempt was made to reach members of the Turkish Society of Hematology. Hematologists who gave their consent and agreed to participate in the study were asked to fill out the questionnaire. The respondents answered questions about their sociodemographic characteristics and their awareness, knowledge, attitudes, and practices regarding the subject of fertility preservation. The participants were informed that the questionnaire would be implemented with full confidentiality and anonymity. 

The questions in the survey were prepared in light of previous studies. These questions probed into the participants’ sociodemographic characteristics and their awareness, knowledge, attitudes, and practices regarding the subject of fertility preservation. The questionnaire was initially administered to 3 hematologists in order to test the comprehensibility of the questions. The validity and reliability of the questionnaire was studied and the internal consistency coefficient of the questionnaire was found to be 0.85. 

The questions were revised on the basis of the feedback received. The study protocol was approved by the local ethics committee. 

**Statistics**

The statistical analysis was performed using version 11.5 of the Statistical Package for Social Sciences (SPSS Inc., Chicago, IL, USA). Descriptive characteristics such as frequency and summary characteristics were calculated for variables of interest. 

## RESULTS

A total of 16 male (64%) and 9 female (36%) hematologists responded to the questionnaire. In the present study, Cronbach’s alpha was found as 0.83. The mean age of the participating hematologists was 47.36±4.32 years. Of the responding hematologists, 4 (16%) worked at private hospitals, 2 (8%) at state hospitals and at training-research hospitals, and 19 (76%) at university hospitals. The characteristics of the participants are presented in Table 1.

All respondents stated their awareness of the possible adverse effects on fertility of the treatment modalities that they were using. Twenty-three (92%) revealed that they had heard of the concept of fertility preservation while 2 (8%) asserted that they had not. The responses of the participants are given in Table 2.

Of the participants, 15 (60%) said that they did not inform patients about fertility preservation options as a routine procedure and 11 (44%) stated that they did not obtain informed consent from the patients or their legal guardians regarding the adverse effects of drugs and/or treatment procedures on fertility. Twenty-three (92%) expressed their feeling that patients and their families should be informed about the subject with the obtaining of written informed consent. On the other hand, 14 (56%) of the participants revealed that their patients or patients’ families knew about and asked whether they could discuss the topic of fertility preservation. 

Of the participants, 23 (92%) said that they did not have a brochure or any other printed materials on the subject of fertility preservation, and they stated that if such documents could be made available, they would have more opportunity to discuss fertility preservation with patients and their partners and families. 

Of the respondents, 19 (76%) said that they did not feel that they had enough information about fertility preservation and, fortunately, 22 (88%) expressed their desire to learn more. 

Among the hematologists participating in the survey, 24 (96%) said that they should consider fertility preservation a priority topic when planning a modality of treatment for patients; only 15 (60%), however, did say that they considered the subject when planning the treatment. 

Of the participants, 17 (68%) professed knowledge about fertility preservation options for male patients and 11 (44%) said that they knew about fertility preservation options for female patients. 

Of the participants, 22 (88%) said that they did not have enough knowledge about oocyte cryopreservation and 13 (52%) reported the same about ovarian tissue cryopreservation. Fifteen (60%) of the participants revealed that they had never recommended sperm cryopreservation to any patients, 19 (76%) stated that they had not recommended ovarian tissue cryopreservation, 20 (80%) stated that there had been no instance where they had recommended oocyte cryopreservation, and, finally, all participants said that they had never recommended embryo cryopreservation. 

Eighteen (72%) of the participants indicated that they had never read any publication on fertility preservation; 20 (80%) stated that they had not read any publication about the subject in the last 6 months. 

Among the respondents, 20 (80%) expressed their approval of patients postponing their treatments for a short period (4 weeks, for example) to accommodate the fertility preservation process. 

Fourteen (56%) of the participants said that the hospital in which their patients were treated had no clinic or assisted conception unit for fertility preservation and 17 (68%) stated that these should be established. Another 20 (80%) said that they had no knowledge about the costs of fertility preservation options. 

Of the participants, 22 (88%) said that they were not familiar with the recommendations on fertility preservation published by the American Society of Clinical Oncology (ASCO) and American Society of Reproductive Medicine (ASRM). Another 24 (96%) admitted that they did not know whether the Turkish Society of Hematology had published a guidebook on fertility preservation, and all participants asserted that the Turkish Society of Hematology should have a guidebook and that they would support its publication and sessions about fertility preservation at congresses of the Turkish Society of Hematology. 

## DISCUSSION

It was found that most of the hematologists in the study were aware that the treatment modalities they used had an adverse effect on fertility. A majority of the hematologists stated that they have heard of the concept of fertility preservation. It was also found that most of the hematologists did not routinely inform their patients about fertility preservation options. Most of the hematologists that participated in the survey stated their lack of adequate knowledge about fertility preservation but, fortunately, most of them expressed their desire to learn more about the topic.

As seen in the results obtained from the survey, a growing number of patients and their families are asking to be informed about the matter of fertility preservation and wish to discuss this with their doctors. It is our belief that it is very important that patients are informed about this subject. The same view has been expressed by ASCO and ASRM [7,8,9]. Patients should be informed and thus made ready to make their own informed decisions. The active participation of patients’ families should also be ensured in sessions where patients are provided information about fertility preservation. This is important because the subject is not only a source of anxiety for the patient but also creates intense feelings in close relatives such as the patient’s spouse.

Most of the hematologists in our study said that they did not routinely inform patients about fertility preservation before the start of treatment. In studies carried out abroad, half of male and female cancer patients were reported as not remembering being informed about fertility preservation [8,9,10]. Those who did remember said that they were not happy with the quality and amount of information that they were given [11,12]. Explaining the adverse effects on fertility of treatment modalities, informing patients about fertility preservation, or referring the patient to another physician for the purpose of obtaining this information is the responsibility of the patient’s attending physician [6].

Because there is no test today to indicate which patients’ fertility will be affected by prescribed treatments and what the impact will be, it is our belief that all patients should be informed about this subject [4]. Fertility preservation alternatives should be explained in detail to all patients and patients should be given the chance to decide. The matter of fertility is an important topic that affects quality of life following treatment. In a study by Schover et al., it was found that 76% of young cancer survivors with no children wished to eventually be parents and, for this reason, were interested in learning about the positive and negative effects of treatment on fertility. In this context, it was also reported that patients were concerned about the negative impact of treatment on a possible pregnancy and on the fetus, should a pregnancy occur [10].

As the majority of the hematologists participating in this study indicated in the questionnaire, there were no brochures or informative booklets available about fertility preservation that could be given to patients to read. Such booklets are generally made available to patients as standard procedure at clinics in Western countries. As the majority of hematologists indicated in the questionnaire in the present study, the availability of this kind of informative printed booklets will enhance the dialog of clinicians and patients on this subject and patients will find increased opportunity to bring up the matter for a more productive discussion. Organizations such as FertileHope [13] in the United States produce brochures of this kind and provide patients with support and guidance.

The majority of the hematologists stated that no clinics dealing with fertility preservation existed in the hospital in which they worked. It was also observed that hematologists were not knowledgeable about the costs of possible fertility preservation options. It may be that the reason a large majority of the hematologists had not referred patients to a fertility preservation center was because they did not know where such centers dealing with fertility preservation were located or the probable cost. It is our belief that associations of professionals in the areas of fertility, hematology, and oncology should collaborate and share information, perhaps forming working committees among themselves if necessary. An oncofertility consortium has been established in the United States [14]. We feel it would be useful for the Turkish Society of Hematology to announce on its website the locations of centers involved in fertility preservation in all regions of the country so that hematologists can easily find the center closest to them.

Another finding in our study was that some hematologists did not consider fertility preservation a priority topic at the time that a course of treatment was being planned for the patient. Other studies on this subject support this finding [15]. Clinicians are more liable to prioritize the discussion of the patient’s potential life-threatening complications. It is, however, true that as effective treatments cause 5-year survival and cure rates to rise, the topic of fertility will increasingly come to the fore as an issue of quality of life [10], and it is imperative that clinicians realize this. Patients and their families are often in a state of shock over life-threatening complications and in such a situation, the subject of fertility options may be completely overlooked or there may be a lack of interest in discussing the matter.

It is very important that hematologists inform their patients in detail about fertility preservation before treatment begins [9]. The information presented to the patient should contain knowledge about the adverse effects of treatment on fertility. The hematologist should provide the patient and the patient’s family with the fertility preservation options and the success rates of fertility preservation modalities, also informing the patient that some options are still in their experimental stages. The knowledge that hematologists impart to their patients should also include information on the costs of fertility preservation options. It is important that the clinician give the patient the address and telephone number of a person or clinic that can be reached and from which information about fertility preservation can be acquired. We also feel that the hematologist’s own coordination with that person or clinic will be important in choosing a fertility preservation option that is suitable to the patient’s clinical status. It is also thought that obtaining written informed consent from the patient or their legal guardians will be important in terms of medicolegal matters.

Based on this study with a limited number of hematologists, it is not possible to specify to what extent these results are generalizable to all hematologists. Thus, we assume that the results of this study can be generalized to a limited degree. Further research with larger numbers of participants should be conducted on the topic.

It is our belief that publishing guidebooks to offer guidance to clinicians about fertility preservation options is important. The respondents to the questionnaire support the publication of such a guidebook by the Turkish Society of Hematology. ASCO and ASRM have made recommendations about fertility preservation in various publications [6,7,8]. The British Fertility Society [16] has also made similar recommendations. Reviewing these recommendations allows clinicians to be more comfortable about this topic and act on it quickly and in coordination. We feel that it is important that a subcommittee that may be formed under the Turkish Society of Hematology prepare a guidebook that will provide hematologists with guidance on this issue.

Most of the hematologists responding to the questionnaire believed that they did not know enough about fertility preservation, but fortunately they exhibited a desire to receive education on this topic. The respondents also supported the idea that the Turkish Society of Hematology should organize a session on fertility preservation at their regular congresses. An initiative of this kind by the Turkish Society of Hematology may be significant in terms of eliminating the lack of knowledge in this area. At the same time, we also think that making the topic of fertility preservation a standard part of the Society’s fellowship education program will be instrumental in enhancing the levels of knowledge. 

## CONCLUSION

Meeting the needs of hematologists for more education and knowledge on the topic of fertility preservation and improving their knowledge, attitudes, and practices in this area is of great importance. The Turkish Society of Hematology may play a significant and key role in such efforts. 

## CONFLICT OF INTEREST STATEMENT

The authors of this paper have no conflicts of interest, including specific financial interests, relationships, and/ or affiliations relevant to the subject matter or materials included. 

## Figures and Tables

**Table 1:**
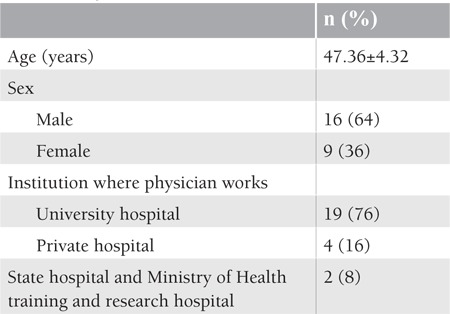
Physician characteristics, n=25.

**Table 2:**
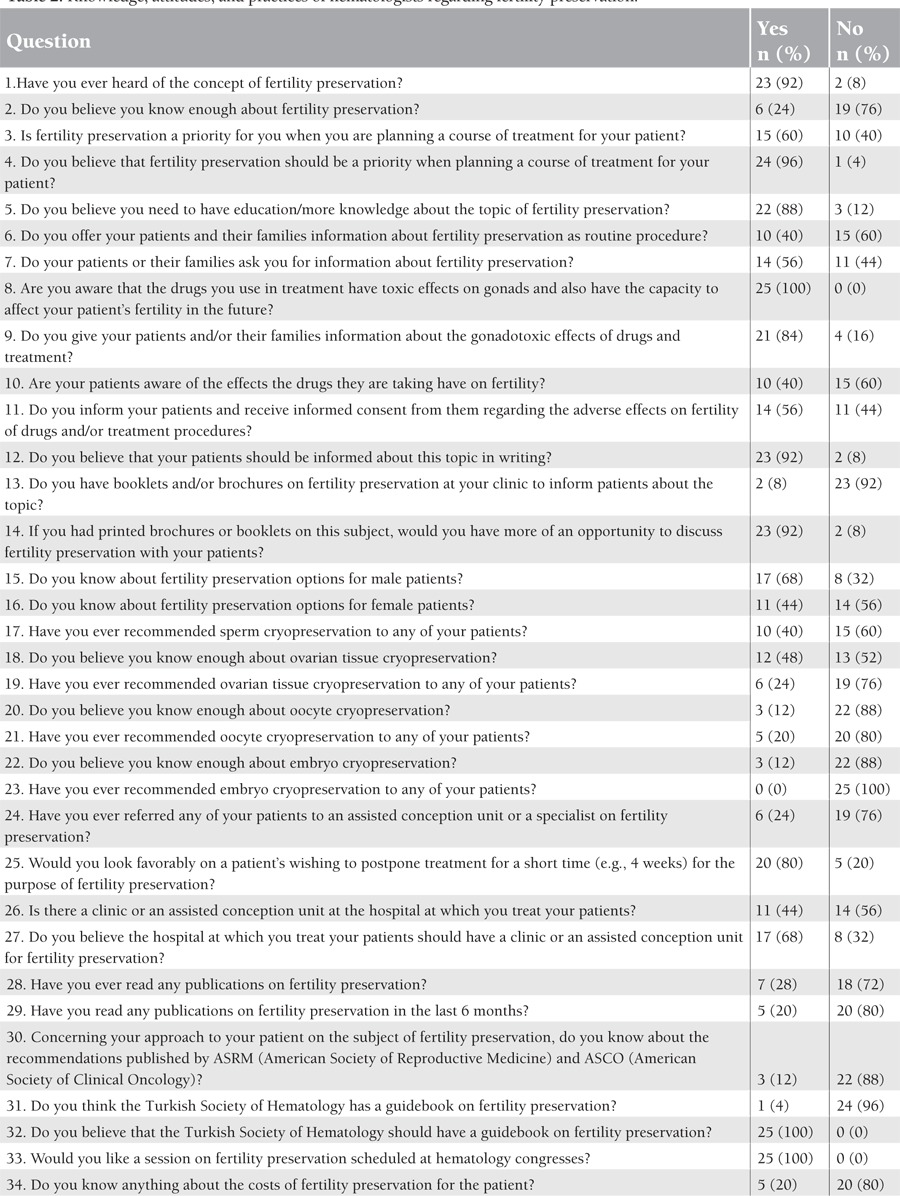
Knowledge, attitudes, and practices of hematologists regarding fertility preservation
